# Teachers’ perceptions of aspects affecting seminar learning: a qualitative study

**DOI:** 10.1186/1472-6920-13-22

**Published:** 2013-02-12

**Authors:** Annemarie Spruijt, Ineke Wolfhagen, Harold Bok, Eva Schuurmans, Albert Scherpbier, Peter van Beukelen, Debbie Jaarsma

**Affiliations:** 1 Utrecht University, Faculty of Veterinary Medicine, Quality Improvement in Veterinary Education, Yalelaan 1, PO Box 80.163, 3508, Utrecht, TD, the Netherlands; 2 Maastricht University, Faculty of Health, Medicine and Life Sciences, PO Box 616, 6200, Maastricht, MD, the Netherlands; 3 University of Amsterdam, Academic Medical Centre, PO Box 22.600, 1100, Amsterdam, DD, the Netherlands

**Keywords:** Seminar learning, Undergraduate (veterinary) medical education, Focus groups, Faculty development

## Abstract

**Background:**

Many medical schools have embraced small group learning methods in their undergraduate curricula. Given increasing financial constraints on universities, active learning groups like seminars (with 25 students a group) are gaining popularity. To enhance the understanding of seminar learning and to determine how seminar learning can be optimised it is important to investigate stakeholders’ views. In this study, we qualitatively explored the views of teachers on aspects affecting seminar learning.

**Methods:**

Twenty-four teachers with experience in facilitating seminars in a three-year bachelor curriculum participated in semi-structured focus group interviews. Three focus groups met twice with an interval of two weeks led by one moderator. Sessions were audio taped, transcribed verbatim and independently coded by two researchers using thematic analysis. An iterative process of data reduction resulted in emerging aspects that influence seminar learning.

**Results:**

Teachers identified seven key aspects affecting seminar learning: the seminar teacher, students, preparation, group functioning, seminar goals and content, course coherence and schedule and facilities. Important components of these aspects were: the teachers’ role in developing seminars (‘ownership’), the amount and quality of preparation materials, a non-threatening learning climate, continuity of group composition, suitability of subjects for seminar teaching, the number and quality of seminar questions, and alignment of different course activities.

**Conclusions:**

The results of this study contribute to the unravelling of the ‘the black box’ of seminar learning. Suggestions for ways to optimise active learning in seminars are made regarding curriculum development, seminar content, quality assurance and faculty development.

## Background

Many medical schools, inspired by social constructivist theories of learning stating that learners should construct their own knowledge in *active* learning environments [1], have embraced small group learning by introducing tutorials, seminars, workshops and group practicals [2]. Small group learning offers students opportunities to discuss and refine their understanding of complex issues, learn how to solve problems and reflect on their attitudes and feelings [3]. Active involvement in questioning, discussion and interaction with subject matter in small groups promotes ‘deep learning’, whereby students elaborate and restructure facts, principles and concepts to build robust cognitive frameworks [4]. Such frameworks are assumed to help students apply what they have learned in new situations [5]. The educational benefits of (active) small group learning can only be realised if certain conditions are met. Dennick & Spencer mentioned organisational (e.g. developing aims and resources), physical (e.g. group size, room layout), psychological (e.g. anticipating group problems) and interpersonal conditions (e.g. setting ground rules) that have to be met for small group interaction to be effective and result in deep learning [4]. According to students, effective learning in groups of fifteen students is promoted by a non-threatening group atmosphere, clinical relevance and integration of questions, and pedagogical materials that encourage independent thinking and problem solving [6]. Other empirical medical educational research has mainly focused on learning in tutorial groups in problem-based learning (PBL) curricula [7]. Due to increasing financial constraints, however, medical and veterinary schools are turning to other formats of active group learning [8], such as seminars and team learning. Since we were unable to find many studies on seminar learning, we conducted a qualitative study to increase our insight into aspects that are conducive to seminar learning.

In this study we use the definition of ‘seminar’ from a study by Jaarsma et al.: a learning session in which a group of some 25 students facilitated by a content expert discusses questions and issues emerging from assigned readings on a topic of practical relevance [9].

A quantitative study of seminar learning by Jaarsma et al. showed that the teacher and the quality of seminar questions were considered to be important factors for seminar learning [9]. An unexpected finding from the same study was that student interactions were not considered to have a positive impact on learning. This finding led to an observational study of the interactions during seminars [10], which showed that the interaction during seminars was dominated by the teacher asking questions to which students gave brief answers, while student-student interactions occurred rarely. Spruijt et al. explored students’ perceptions of seminar learning and found that apart from the teacher and the quality of seminar questions, the quality of the preparation materials, the course schedule and the alignment of the educational methods within a course impacted on the effectiveness of seminar learning. Students also emphasised the importance of the teacher’s didactic approach, group composition and size, the interaction and atmosphere in the group and representative assessment [11].

Because of the importance of their contribution to seminar learning, and because they are likely to have a different perspective on seminar learning compared to students, we sought teachers’ perceptions of seminar learning in the expectation that their views would add to our understanding of the ‘black box’ of seminar learning.

We conducted a qualitative study to explore which aspects teachers consider to be important for seminar learning.

## Methods

### Educational setting

The study was conducted at the Faculty of Veterinary Medicine, Utrecht University, the Netherlands (FVMU), which offers a three-year bachelor programme and a three-year master programme. Each year 225 new bachelor students are admitted. The systems-based bachelor curriculum focuses on basic science knowledge, clinical science knowledge and practical skills. Of the total study time, 30-40% is devoted to lectures, seminars and practicals, while the remaining time is designated for preparation for educational sessions and assessments. Assessment consists mainly of written end-of-course exams.

Seminars attended by groups of around 25 students are the dominant educational method, taking up 40-60% of student-teacher contact time. The objective of seminar learning is to promote active and deep learning by providing interactive student-centred sessions in which students are challenged to discuss questions and issues relating to subjects of practical relevance [9]. Students prepare for seminars by reading assignments with questions, which are published in the study guide. Several two-hour seminars, facilitated by different content experts, are offered every week (depending on the course schedule). Attendance is optional. Students are required to prepare for seminars but are free to do so individually or in a group. Except for duration (2 hours maximum) and the maximum number of students (25), there are no detailed teacher guidelines on procedural matters and teacher guides are mostly limited to a description of seminar content. Student groups remain together for the duration of a semester, but teachers vary depending on seminar content. Teaching faculty attend a two-year faculty development programme, which includes personal mentoring. Quality assurance is based on course evaluations.

### Data collection method

As the aim of the study was to increase understanding of seminar learning we explored teachers’ views concerning seminar learning in a qualitative focus group study. This method relies on group interaction to generate rich data about relatively unexamined issues [12] and it can ‘give rise to a synergy that is lacking from individual interviews’ [13]. Since we aimed to elicit a wide variety of opinions, we considered focus groups to be the most appropriate method for our study.

### Participants

The principal researcher sent an email to all the teachers who had facilitated two or more seminars in the academic year 2010–2011 (N=174), inviting them to participate in a focus group study about seminar learning and offering a small reward for participation. Of 174 teachers that were eligible for inclusion 78 did not respond, 47 were unable to participate due to time constraints and other priorities and 25 declined to participate because they could attend only one session. The 24 teachers who were willing to participate on the proposed dates were assigned to one of three groups depending on their time preferences.

Each group took part in two sessions of around ninety minutes with a two-week interval (Table 1). Twenty-one participants were veterinarians and three were biologists. Eight participants (33%) were clinicians. Due to illness, two participants attended only the first session.

**Table 1:** Overview of participants per focus group

**Focus groups (FG) number**	***N *First session**	***N *Second session**
FG 1	11	11
FG 2	7	6
FG 3	6	5

### Procedure

At the start of the first session, the researcher who moderated all the sessions (IW) assured the participants that confidentiality was guaranteed and asked them to sign an informed consent form. The focus of the first session was elicitation of aspects that influence seminar learning, and these aspects were elaborated upon during the second session. All sessions were semi-structured through pre-defined open questions based on findings from earlier research [11] (Table 2). The questions were new to the participants and the moderator asked additional questions if answers to the questions required clarification. After each session, the moderator summarised the key points and asked for verification. One week after each session, the participants received a summary with a request for corrections and comments for respondent validation [14]. As no new aspects emerged after three groups had participated in two sessions, data collection was stopped.

**Table 2:** Interview questions


**Session 1**	What are the goals of seminar learning?
	What aspects influence seminar learning?
	What are reasons why seminars do not always work?
**Session 2**	What is the role of the seminar teacher?
	What makes for an effective seminar teacher?
	What makes for a safe learning climate?
	What makes for effective preparation to improve seminar learning?
	What would the ideal schedule for effective seminar learning look like?
	What makes for effective seminar questions?
	What can be done to activate the students during the seminar?
	What would you recommend to enhance seminar learning?

### Data analysis

The main objective of the analysis was to interpret the data in order to arrive at themes and categories that shed light on how seminar teachers perceive seminar learning.

In order to gain a good general idea of the data, the first author (AS) listened to the tapes of the first sessions and read all the transcripts. She then wrote a preliminary descriptive summary of each session and discussed these with the moderator (IW) until consensus was reached. As stated earlier, all participants were asked to comment on the summary of their session. The analysis of the first session gave rise to the questions for the second session. The first step of the analysis was repeated for the data from the second sessions.

Using software for qualitative data analysis (Atlas TI), the first author used a latent thematic analytical method to identify, analyse and report patterns (themes) in the data set [15] of all the tapes and transcripts. In latent thematic analysis, the development of themes involves interpretative work [15]. The analysis is conducted from a constructionist point of view and involves an iterative process of reducing and displaying the data, culminating in a scheme of codes. The authors used an inductive approach in analyzing the interviews. In order to enhance the reliability of the analysis, another researcher (ES) independently read and coded 20% of the transcripts. Discrepancies between initial coding schemes were reconciled through discussion and the final coding scheme was established. Based on their relationships and connections, the codes were categorised and AS and ES used the categories to independently re-analyse the content of 20% of the transcripts, which resulted in two additional codes. The code categories were interpretively sorted into potential key aspects and sub-aspects. These aspects were reviewed, defined and named in an expert meeting of the first author and four experts in (veterinary) medical education (co-authors: IW, AS2, PvB, DJ). Finally, quotes were selected to illustrate the findings (Table 3).

**Table 3:** Examples of transcript analysis

**Quote**	**Initial code**	**Coding category**	**Key aspect**
*“Questions used in seminars should provoke discussion”*	Quality seminar questions	Seminar questions	Seminar goals and content
*“The preparation materials have to be of value to the seminar.”*	Suitability of preparation materials for seminar	Preparation materials	Preparation

### Ethical considerations

The study was approved by the ethical review board of the Netherlands Association for Medical Education (NVMO-ERB). Participation was voluntary and participants were assured of confidentiality. All participants gave written informed consent. The participants were assured that they were free to leave a session or not answer a question if they desired to do so.

## Results

Participants’ comments were clustered into seven key aspects that influence seminar. In this section, these key aspects are described in detail and illustrated by quotes. To elucidate, we have clustered the key aspects in three overarching themes: teacher, student and organisation.

### Teacher

#### Seminar teacher

According to the participants, teachers have an important role in seminar learning. An effective seminar teacher should have expertise in the seminar subject, should not be perceived as threatening by students and have good interpersonal and didactic skills to stimulate interaction. It was also said that seminar teachers should provide context and examples to clarify subjects and help students identify gaps in their knowledge. Another task was to generate enthusiasm for their subject among students and ensure that learning objectives were reached.

*“A seminar teacher has to be enthusiastic. It would be ideal if he could accurately gauge students’ level of knowledge, is aware of the place of the seminar in the course and of the learning objectives of the course and, actually, of the whole curriculum.”* (FG 3)

Participants emphasised the importance of the teachers’ role in education. Some seminar teachers were full-time teachers, while others were involved also in research and patient care. This latter group experienced some difficulty estimating the knowledge level of students, because they infrequently meet the students.

Seminar learning was also affected by the extent of teachers’ involvement in seminar development (degree of ‘ownership’):

*“I think seminars I facilitate are more effective when I have constructed them myself or together with my colleagues. Then I know why I set that specific question and I feel more responsible for the seminar I think. Besides, I can see what does not work during the seminar, so I can change it for the next time.”* (FG 3)

Participants recommended improving collaboration between seminar teachers within and across disciplines to improve seminar quality.

*“We have an integrated curriculum, so I think we should promote integration by more collaboration between disciplines in developing a seminar.”* (FG 2)

### Student

#### Students

Seminar learning depends for a large part on students’ motivation, preparation and participation during the seminar, teachers said. They agreed that it should be clear to the students that a seminar can only be effective if students actively engage with the subject. Students should also know what is expected from them during a seminar.

*“I think students should be more aware of their role in the seminar. Students are not always very well prepared; I really think some of them need to work on their time management skills.”* (FG 1)

According to the participants seminars are most effective when students participate actively and are willing to collaborate with other students, are interested in the subject and curious to know more than just the answers to the questions. Teachers suggested that active student participation could be promoted by providing context and addressing students by name. It was also considered important that passive students should not be rewarded for their inactivity. Students’ learning style was also of influence on seminar learning some teachers thought:

*“I feel that seminars work well for students who have a specific learning style, but other students just want to listen and not participate actively. It’s very hard to make a seminar interactive that way.”* (FG 1)

#### Preparation

Student preparation was considered to be essential for effective seminar learning.

*“Sometimes it is not possible to have in-depth discussions, because students have not prepared properly, and consequently do not know enough about the subjects and/or are unable to discuss it.”* (FG 2)

While some teachers said that poor preparation was due to students’ lack of motivation or poor time management skills, others stressed the importance of high quality preparation materials. Materials should be clear, with appropriate preparation questions and in line with and of value to seminar content. Most teachers agreed that the amount of preparation materials should be in proportion to the amount of time available for preparation, but they also indicated that they had little idea of the amount of time involved.

*“We have guidelines for the amount of preparation materials we can assign for a seminar, but the guidelines are difficult to follow, as they say that students can read 4 or 6 pages an hour. Besides, I’m only responsible for one subject in a course and I honestly have no idea how much preparation time students have or need for the entire course.”* (FG 3)

Some teachers thought that effective preparation could be promoted by scheduling preparation time for students, but other participants disagreed:

*“I think scheduling preparation time is childish and not appropriate in an academic environment. Students at this university should be able to manage their time properly. In fact, even assigning preparation materials for a seminar is unfitting in an academic course; students should search for preparation materials themselves.”* (FG 2)

Teachers said that the absence of standard sanctions if students do not prepare for sessions affects students’ preparation behaviour. Teachers have different ways of coping with unprepared students, so students do not know where they are if they haven’t prepared.

*“It is hard to determine whether a student has not prepared for the seminar or whether they have not interpreted the materials correctly, so it is not easy to take action. It might be an option to assess the students during the first 5 minutes by means of a short written test, but that would mean a lot of work for us.”* (FG 3)

Teachers also talked about their own preparation time. Teacher preparation time for a seminar was considered essential for junior teachers but not for specialized clinicians. Most teachers preferred facilitating the same seminar several times during a course.

*“For me it is essential that I can facilitate one seminar several times, so I will be able to try different didactic approaches and evaluate their effects. And, of course, it is more efficient for my own preparation. I think facilitating the same seminar 3 or 4 times would be ideal… If I have to facilitate the same seminar more than 4 times, I don’t know what I have said to which group anymore.”* (FG 1)

Teachers proposed that time and money be invested in producing clear and suitable preparation materials for the seminars, for example digital self-study tools.

*“I think we should make some of the preparation materials more fun and more modern, for example by using e-learning modules. Unfortunately I have neither the time nor the money to prepare such materials.”* (FG 2)

#### Group functioning

*“A non-threatening group atmosphere and active student participation are most important in seminar learning if you ask me. If everyone respects each other and wants to participate, group discussions will arise and learning will be effective.”* (FG 1)

Teachers indicated that group size, continuity of group composition, teacher and student behaviour and mutual respect contributed to a safe learning climate.

*“A safe learning climate can be enhanced by the introduction of group teachers and by ensuring that a teacher and a group of students can stay together for the duration of a course. Students and teacher can get to know each other better, students will feel safer, so more discussion will take place and students can elaborate on their understanding of the materials.”* (FG 3)

*“I usually bring a participant list to the seminar, so I can address the students by name. I really think that students are more attentive and participate more actively, when they feel they are not anonymous.”* (FG 3)

It was recommended to reduce group size to decrease anonymity and help students and teachers build a positive relationship. The teachers acknowledged that smaller groups would mean more seminars on the same subject and consequently more teaching time. Adjusting the facilitating method within a seminar or reducing seminar time and content were proposed to resolve this problem.

*“I think learning in a group of 25 students can be effective when you adjust the facilitating method to the size of the group, for example by dividing the group into five subgroups to create a safe learning climate.”* (FG 1)

### Organisation

#### Seminar goals and content

Teachers mentioned multiple general goals of seminars:

*“A seminar has several goals if you ask me. It allows students to actively play with the seminar subject and discuss difficulties with each other and it is an opportunity to ask us questions.”* (FG 2)

Other goals were: applying seminar content to clinical situations, dealing with uncertainty and problem-solving and presentation skills. Furthermore, seminars helped students to structure the important aspects in the learning materials.

In the discussion of seminar goals, the teachers also talked about the suitability of subjects for the seminar method.

*“Occasionally when I’m facilitating a seminar I think that the subject would be more suitable for another educational method, such as a practical, lecture or an e-learning module.”* (FG 3)

Participants emphasized the significance of the relationships between seminars and between seminars and other educational methods used during the course. They said that there should be a clear connection between seminars and other educational methods. In addition to proper alignment and structure of subjects within a course, seminars had to be well structured and integrated. At the start of a seminar, the specific seminar goals should be explained and the seminar should end with an overview of seminar content. Seminar questions should increase in difficulty.

In discussing specific seminar content, the teachers placed great importance on the quality and amount of seminar questions. According to the teachers, effective seminar questions were clinically relevant, academically challenging and clearly defined. They encouraged problem solving and discussion, promoted a deeper understanding of the subject and helped students to apply what they had learned in similar situations in the future.

“*Sometimes the seminar questions do not stimulate discussion, because they only ask for factual knowledge.” (*FG 1)

Some teachers thought that ‘factual knowledge questions’ should be avoided entirely, while others thought they could be used as introductory questions if they were relevant to the other questions.

*“Good seminar questions are not questions that can be answered by copying sentences from a book.”* (FG 3)

Teachers mentioned that content overload due to too many pre-set questions hampered effective discussions.

*“Sometimes students stop discussing a question because they are afraid that there will not be enough time to discuss all the other questions.”* (FG 3)

The introduction of an evaluation tool for use after each seminar was recommended by participants to provide feedback to teachers on the quality of the preparation materials and seminar content.

#### Course schedule and course coherence

According to the teachers, seminar learning was affected by the sequencing of educational methods. The main subjects should be introduced in a lecture after which students should have time for preparation followed by discussion in a seminar.

*“Last week, I had to facilitate a seminar on a subject that had not been introduced in a lecture, due to scheduling problems students say… As a result it was impossible to have an in-depth discussion.”* (FG 1)

Too much contact time on one day was thought to hamper active participation in seminars. Additionally, the distribution of contact time over the course was considered to have an impact on seminar learning. The teachers recommended analysing course schedules.

*“I think students suffer from peak workload in some weeks, which causes students to say it was impossible to prepare for a seminar because there just wasn’t enough time.”* (FG 2)

The teachers noted that at the end of a course, students gave priority to preparing for the exam over preparing for seminars.

Some teachers thought seminar teaching would be optimised if they could facilitate several different seminars in one course, as this would enable them to show students how different topics are connected and to refer to previous seminars or preparation materials. They recommended course and curriculum mapping, so teachers and students can easily see the connections between different educational methods within a course and between courses.

*“It is important that we critically appraise our course materials and think about which subject suits which educational method to make all the different educational methods within a course more effective.”* (FG 3)

#### Facilities

Participants emphasised the importance of well-functioning audio-visual equipment (computer, beamer), the presence of whiteboards and appropriate classrooms with ventilation, windows and light to help students stay concentrated. They also said that different seating arrangements should be possible, requiring classrooms with enough space and small tables.

Table 4 shows the key and sub-aspects affecting seminar learning which were derived from the transcripts.

**Table 4:** Aspects influencing seminar learning emerging from the focus groups

**Themes**	**Key aspects**	**Sub aspects**
Teacher	Seminar Teacher	• Teacher role and involvement in education in general (full time teacher vs. clinician with educational tasks)
		• Pedagogical skills/pedagogical degree
		• Interpersonal skills
		• Subject knowledge
		• Role of the teacher in a seminar
		• Experience in facilitating seminars
		• Ownership of the seminar
		• Collaboration with other course teachers
		• Being motivated for seminar teaching
		• Amount of time for seminar preparation
		• Work load
Student	Students	• Student goals
		• Student roles
		• Student motivation
		• Student behaviour
		• Student learning styles
		• Student collaboration skills
		• Student time management skills
	Preparation	• Materials (Clarity, Amount, Difficulty, Guiding preparation questions, Type)
		• Preparation time
		• Suitability of preparation materials for seminar
		• Scheduling of seminars in relation to other educational methods & assessment
		• Sanctions for not preparing (Group, Teacher)
		• Teacher’s expectations of students
		• Student characteristics (Motivation, Prior Knowledge, Interest in subject, Extra-curricular activities)
	Group functioning	• Size
		• Group composition
		• Amount of interaction
		• Safe learning climate (continuity of teacher-student group combinations; respect)
		• Facilitating method used within seminar
		• Room (physical space, seating arrangement)
	Seminar goals and content	• General seminar goals (clarity)
		• Specific seminar learning objectives (clarity)
		• Suitability of subject for seminar
		• Role of seminar in course
Organisation		• Relation and integration of different seminars in a course
		• Integration within seminars
		• Availability of seminar materials
		• Quality and type of seminar questions (provoking discussion, clinically relevant, academically challenging)
		• Number of seminar questions
	Course schedule and coherence	• Sequence and coherence of different educational methods
		• Distribution of contact time over the course
		• Amount of contact time per day
		• Planning of the assessment
		• Efficient planning of number of seminars per teacher (<3-5)
		• Distribution of time of teaching staff between patient care, research and teaching
	Facilities	• Availability of markers & whiteboard
		• Availability of audio-visual materials
		• Climate & Light
		• Seating arrangement

## Discussion

Focus groups with teachers revealed seven key aspects that were thought to influence seminar learning: the seminar teacher, students, seminar goals and content, facilities, group functioning, preparation and course schedule and coherence.

The teachers’ views appeared to be quite consistent with students’ perceptions of seminar learning [11] and with aspects affecting small group learning described in other studies [3-7,16-19]. Organisational, physical, psychological and interpersonal prerequisites for small group interaction aimed at promoting deep learning were also confirmed by our findings. The difficulty in creating favourable interpersonal conditions that was mentioned by the teachers may be due to the combination of fairly large group size and variable group composition. The results showed that teachers’ views and those of the students [11] differed, particularly with regard to ‘seminar goals and content’. Teachers emphasized the significance of the suitability of subjects for seminar learning and of clear seminar goals, while students did not question the selection of seminar subjects but focused on the structure of seminars and methods used to facilitate learning. Whereas teachers scarcely discussed various facilitating methods within seminars, students stressed the value of dividing the group in subgroups [11]. Kooloos et al. also found that students preferred working together in smaller subgroups within a group of 15 students [20]. We would recommend faculty development programs to focus on different facilitating methods.

Teachers and students agreed on the importance of the amount and quality of seminar questions. Seminar questions should be clear, encourage problem solving and discussion, and be clinically relevant and academically challenging. This confirms Steinert’s findings regarding effective questions in small group learning [6]. According to the teachers, effective questions stimulate a deeper understanding of subjects and help students to apply what they have learned when they encounter similar situations in the future.

Another interesting finding is that the teachers – contrary to our expectations – paid no attention to assessment as a driver of seminar learning, but only referred to the negative effect of assessment on students’ preparation for seminars at the end of the course when the course exam was approaching. This raises questions about the relationship between seminar content and the end-of-course exams.

Teachers’ views on seminar goals were consistent with the goals of the school [9] and the goals for small group learning reported by Crosby & Hesketh and Steinert [5,6]. The teachers’ perceptions of the role of the teacher in seminar learning were similar to what has been reported for the teachers’ role in tutorials, which requires active listening, questioning, facilitating, responding, giving feedback and providing structure [4,6]. In this respect, however, there seems to be a discrepancy between the teachers’ views and findings from Jaarsma’s observational study, which revealed that in some seminars the teacher was responsible for 93% of the interactions [10]. This suggests that teachers may (can) not always live up to their own expectations regarding their role performance.

A final important finding is that the teachers focused not only on effective seminars during the focus groups but also on (the relationship with) other educational methods used in the course. Some seminars seemed to ‘stand by themselves’ and teachers felt that they were ‘flown in’ and had missed the connection with the rest of the course and the other teachers. This suggests a need for a ‘culture of education’. A possible explanation may be that in certain disciplines, staff has clinical duties in addition to tasks in teaching and research. The teachers articulated a need for curriculum mapping to help them see their role in the bigger picture of the curriculum. This supports Harden’s support for the importance of curriculum mapping for an effective integrated curriculum [21]. The teachers made concrete recommendations for quality management and faculty development to improve seminar learning, focussing on the importance of partial ownership of seminars and teacher-teacher collaboration.

### Limitations

A limitation of this study is the low response rate: 24 out of 174 teachers. It is possible that it was the more ‘education minded’ teachers that participated and their views may not reflect those of the majority of teachers. Another limitation is that the study was conducted at one university; comments may be context dependent, so it is not clear to what extent the findings are transferable to other settings [14]. Individual interviews and observations could have enriched the data and validity of this study.

### Future research

The results of this study suggest areas for further research, such as preparation materials, seminar evaluation, course coherence and sequencing of the different educational methods. It seems worthwhile to investigate whether optimising these aspects could have a beneficial effect on seminar learning.

## Conclusions

The results of this study contribute to the further unravelling of the ‘the black box’ of seminar learning initiated in earlier studies. The seminar teacher, students, preparation, group functioning, seminar goals and content, course coherence and schedule and facilities were identified as key aspects influencing seminar learning. Important sub-aspects were teachers’ involvement in seminar development (‘ownership’), the amount and quality of preparation materials, a non-threatening learning climate, continuity of group composition, suitability of subjects for seminar teaching, the number and quality of seminar questions, and alignment of course activities. The results give rise to concrete tips for curriculum development, quality assurance, faculty development and seminar content. We think seminars can offer a good alternative to other small group formats provided conditions for students’ active participation and interaction are fulfilled.

### Practical implications

The results suggest that seminar learning would benefit when quality management and curriculum review focused on:

• Evaluation of the amount, clarity and suitability of preparation materials;

• Evaluation of seminar content and seminar teachers, in particular the number and quality of seminar questions and of the performance of seminar teachers;

• Introduction of facilitating methods aimed at stimulating active participation and interaction in seminars, such as buzz groups;

• Curriculum mapping and course evaluation: critical review of course content with attention for overlapping subjects and matching educational methods to content. Course schedules and alignment and coherence of different educational methods should be analysed;

• Creating a positive educational culture by allocating more time and rewards for teaching tasks;

• Creating co-ownership of seminars and teacher-teacher collaboration so teachers can enhance their pedagogical skills in seminar learning.

## Appendix

Appendix 1: Page from study guide of the course ‘Digestion’ from Bachelor Veterinary Medicine of Faculty of Veterinary Medicine, Utrecht University (2012) [[22]].

### Content seminar 5 ´Exocrine pancreas insufficiency and bile´

During this seminar a couple of aspects of the general reaction pattern and function of the exocrine pancreas, and the digestive function of bile is discussed.

#### Preparation materials

• Reading ‘Syllabus Digestion’ paragraph 7.2.7 about diseases of the pancreas and the digestive function of bile;

• Reading ‘Dukes’ physiology of domestic animals, Reecee (12th edition), chapter 25: Secretions of the stomach and accessory glands, paragraph ‘Biliary tract’, p 415–417.

#### Preparatory questions

• Describe the histologic structure of the pancreas;

• Which enzymes carry out the intraluminal digestion?

• What is the function of bile in the intestine?

#### Example of some seminar questions from this seminar

A) Casus 1: A 5 year old, male castrated Bull terrier has symptoms of icterus and vomiting since 4 days. During the abdominal ultrasound a corpus alienum is diagnosed in the proximal part of the duodenum.

1. How can you explain the dogs’ complaints?

2. Describe the possible changes of fats and fatty acids in the faeces of a patient with intra- or extra hepatic bile duct obstruction;

3. How can you explain the ‘concrete coloured’ (grey) faeces of this dog?

B) Explain what effects are to be expected of the presence of exocrine pancreas insufficiency on:

• Plasma glucose concentration;

• Serum protein concentration;

• Haematocrit.

## Competing interests

The authors declare that they have no competing interests.

## Authors’ contributions

All authors made substantial contributions to the study conception and design. IW conducted the focus group interviews. AS analysed and interpreted the data in a process of collaboration with HB, ES, IW and DJ. AS2 and PvB provided advice on the method and contributed to the data interpretation and discussions. AS wrote the first draft of the article. All authors contributed to the subsequent critical revision of the article. All authors read and approved the final manuscript.

## Authors’ information

Annemarie Spruijt, DVM, is lecturer and PhD-candidate in Quality Improvement in Veterinary Education at the Faculty of Veterinary Medicine, University of Utrecht. Her research interests include seminar learning, faculty and curriculum development.

Ineke Wolfhagen, PhD, is Associate Professor and Educational Psychologist at the Faculty Health, Medicine and Life Sciences, Maastricht University. Her research interests include problem-based learning, teacher evaluation and quality assurance.

Harold Bok, DVM, is lecturer and PhD-candidate in Quality Improvement in Veterinary Education at the Faculty of Veterinary Medicine, University of Utrecht. His research interests include work-based learning and assessment.

Eva Schuurmans, DVM, is Research assistant at Quality Improvement in Veterinary Education at the Faculty of Veterinary Medicine, University of Utrecht. Her research interests include the undergraduate curriculum organisation.

Albert Scherpbier, MD, PhD, is Professor and Dean at the Faculty Health, Medicine and Life Sciences, Maastricht University. His research interests include quality assurance and professionalization of medical education.

Peter Van Beukelen, DVM, PhD, is Professor Quality Improvement in Veterinary Education at the Faculty of Veterinary Medicine, University of Utrecht. His research interests include quality assurance, faculty development, professional conduct and workplace-based learning.

Debbie Jaarsma, DVM, PhD, is Professor in Evidence-Based Education at the Academic Medical Centre, University of Amsterdam. Her research interests include faculty development, seminar learning and curriculum development.

## Pre-publication history

The pre-publication history for this paper can be accessed here:

http://www.biomedcentral.com/1472-6920/13/22/prepub
